# Occult Vascular Transection Identified by Point-of-care Ultrasound Demonstrating Evidence of Retrograde Flow

**DOI:** 10.5811/cpcem.2019.7.42808

**Published:** 2019-09-30

**Authors:** Nadia Aracelliz Villarroel, William Wagner, Elizabeth Schoenfeld

**Affiliations:** UMASS Medical School-Baystate Medical Center, Department of Emergency Medicine, Springfield, Massachusetts

## Abstract

Acute vascular injury can be a cause of significant disability and morbidity. High clinical suspicion and a thorough physical examination are key components to facilitate a timely diagnosis. We present a case of acute vascular injury after isolated penetrating trauma. Physical examination demonstrated a strong distal radial pulse; however, point-of-care ultrasound facilitated an evaluation of the directionality of arterial flow, demonstrating that flow was retrograde via the palmar arch. We subsequently identified a proximal and complete arterial laceration.

## INTRODUCTION

Early diagnosis of acute vascular injury is critical to expedite treatment interventions and ultimately improve patient outcomes and prevent disability and amputation. A complete physical exam and a high index of suspicion are pivotal for early identification of an acute vascular injury. When “hard signs” are present clinical consensus mandates immediate surgical intervention.^1^ “Hard signs” of vascular injury include absent distal pulses, limb ischemia, pulsatile flow, hemorrhage with shock, or an expanding hematoma. Alternatively, the presence of “soft signs” of vascular injury (such as significant hemorrhage reported at the time of the injury, diminished but palpable pulses, a stable hematoma, a peripheral nerve deficit, or anatomic proximity of the wound to a major artery) should prompt further diagnostic testing.^1^

Point-of-care ultrasound (POCUS) is an accessible tool in the emergency department (ED) that can assist in the diagnosis of an acute arterial injury. Previous studies demonstrate how ultrasound aids in the diagnosis of an acute arterial injury through the identification of the presence or absence of flow, the presence of pseudoaneurysms, abnormalities in waveform and flow velocity, and the quality of the Doppler signal.^2^,^3^,^4^,^5^ When using ultrasound for a suspected arterial injury, it is also important to consider directionality, as strong collaterals can generate retrograde flow and mimic a normal exam. We present a case of acute vascular injury after an isolated, small penetrating trauma to the forearm. The patient presented with “soft signs” and normal findings. Using POCUS and accounting for directionality, we were able to demonstrate an occult arterial injury, which was masked on physical examination by strong collateral flow.

## CASE REPORT

A 30-year-old previously healthy male presented to the ED with a laceration to the left forearm. The injury occurred just prior to arrival when the patient was carving a Halloween pumpkin with a large steak knife, which slipped and punctured the volar aspect of his left forearm, halfway between his elbow and his wrist. His wife noted projectile bleeding immediately after the injury. Emergency medical services arrived and applied a tourniquet proximal to the injury of the affected limb. Upon arrival to the ED, the tourniquet was removed. On physical examination “oozing blood flow consistent with venous injury” and no pulsatile flow was present. He reported 5/10 pain over the area of the wound. He denied numbness, paresthesias, decreased range of motion, or change in skin color.

The patient had no significant past medical history and was not on any medications. The vital signs were as follows: temperature of 98.9° Fahrenheit; pulse of 80 beats per minute; blood pressure of 140/79 millimeters of mercury. Physical examination was significant for a 1.5 centimeter (cm) full thickness laceration to the volar aspect of the left forearm, with slow pooling of blood in the wound. Radial pulses were 2+ distal to the laceration. There was no pulsatile bleeding, and bleeding was well controlled with pressure to the wound. Sensation was grossly intact, capillary refill was less than two seconds, and motor strength – including grip, flexion and extension of wrist and all digits, and abduction and adduction of digits – were intact. The skin was normal without mottling or pallor.

**Image 1:**
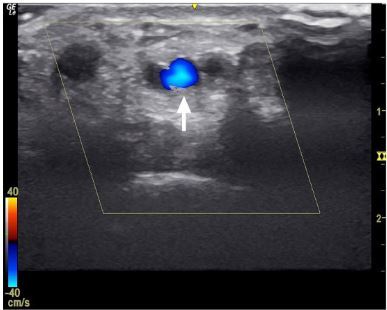
The image exhibits the ultrasound of the radial artery distal to the injury in transverse view. The white arrow demonstrates the radial artery, with color Doppler over the vessel. The blue color within the radial artery signifies retrograde flow, with blood flow in the direction away from the ultrasound probe.

The patient’s blood work in the ED was notable for a mild anemia hemoglobin of 12.9 grams per deciliter (g/dL) (reference range 13.7 – 17.1 g/dL) and hematocrit of 41% (40.5–50.0%) and mild thrombocytopenia of 148 thousand cells per cubic millimeter (k/mm^3) (reference range 150 – 460 k/mm^3). We performed a POCUS with the 10-megahertz linear array transducer to evaluate for potential arterial injury. Using color Doppler, the POCUS demonstrated pulsatile flow in the radial artery. However, when the directionality of the flow was evaluated, POCUS confirmed the suspicion for a vascular injury by demonstrating retrograde flow in the radial artery at the level of the wrist crease (Image 1 and Video).

**Image 2:**
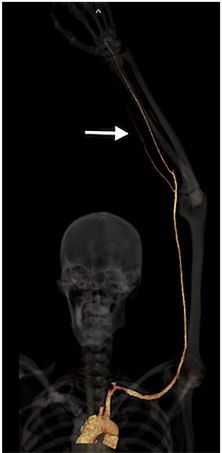
The image depicts the computed tomography angiography reconstruction with bone shadow. The white arrow demonstrates the vascular cutoff of the radial artery in the area of the soft-tissue laceration.

Vascular surgery was consulted and requested computed tomography angiography (CTA) of the extremity for operative planning. The CTA confirmed the ultrasound findings, demonstrating a focal cutoff of the radial artery at the level of the laceration with a patent ulnar artery providing retrograde perfusion to the distal radial artery (Image 2). The patient was taken to the operating room immediately from the ED for intra-operative exploration, which revealed a complete radial artery transection. After repair of his radial artery, the patient was hospitalized, placed on a heparin drip for 24 hours, and given clopidogrel and aspirin. On postoperative day two a duplex ultrasound demonstrated patency of the radial artery and the patient was discharged.

## DISCUSSION

While this patient presented with a clinical history of pulsatile bleeding concerning for an arterial injury, upon arrival to our ED there were no “hard signs” of vascular injury. Historically, a clinical suspicion for vascular injury was determined by the presence of “hard” or “soft” signs of vascular injury. Previous literature has mandated surgical exploration of a wound for “hard signs.”^1^ However, the recommendations for “soft signs” are more nebulous.^1^ In the absence of “hard signs,” many physicians opt for a CTA to diagnose arterial injuries; however, CT carries potential risks in the form of radiation and allergic reactions. An alternative to this is ultrasound.

Rad et al.^3^ compared CTA to ultrasound with Doppler waveforms examined at multiple locations along the affected limb of patients with concern for an arterial injury. If there was a waveform abnormality noted at any distal locations, the expert radiologist performing the study was then prompted to more closely evaluate the entire artery. This technique resulted in a sensitivity of 94.8% and a specificity of 91.6%. One false negative was attributed to a thick dressing that wasn’t removed. Importantly, a second arterial injury was missed due to strong collateral blood flow that masked the injury, consistent with the clinical presentation of our patient.

Wani et al.^4^ compared CTA to ultrasonography in patients with “soft signs” concerning for vascular injury. Patients were evaluated with ultrasound performed by a radiologist, and if positive, the patients were surgically explored. However, if the ultrasound was negative, patients were evaluated via CTA. A total of 150 patients underwent ultrasound, and 110 demonstrated positive findings and were taken for vascular surgery. Of the 40 patients who had no ultrasound findings, seven were found to have CTA findings concerning for vascular injury. The authors reported a sensitivity of 94% and a specificity of 82.5%.

Only one study to date has evaluated the use of POCUS to identify acute traumatic vascular injuries. Monoforano et al.^6^ examined the accuracy of a focused Doppler ultrasound protocol in the rapid assessment of arterial injuries after penetrating trauma. Two board-certified physicians with specializations in ultrasound performed a two-point Doppler assessment of the posterior tibial and dorsal pedis arteries with POCUS, specifically assessing for the presence of flow and characteristics of the Doppler waveform. A pathologic waveform was defined as absent flow or a biphasic or a monophasic waveform in one of the examined arteries. Of the 149 limbs included in the study, 134 were correctly identified as having no acute injury while 15 limbs were correctly identified as having an acute arterial injury, first by standardized full color Doppler and then by CTA. The study found that through utilization of these methods and the implementation of a two-point Doppler protocol, a sensitivity of 100% and specificity of 100% with a positive predictive value of 100% could be achieved.

Not all studies of ultrasound have demonstrated such accurate test characteristics. In one such study the specificity was quite high at 99%, but the sensitivity was low at 50%.^7^ The ultrasound studies were performed by either vascular technicians or a vascular surgery fellow specializing in vascular ultrasound. The authors noted, however, that both of the missed arterial injuries were small pseudoaneurysms. Furthermore, they attributed this low sensitivity to a low prevalence, as in this study only four injuries were found on angiography, and two of them were missed on ultrasound for a sensitivity of 50%.

A recent meta-analysis by DeSouza et al.,^8^ however, appears to support ultrasound as a viable and accurate option. The authors examined a large database of studies that included ED patients with penetrating extremity injuries. Among those included in the meta-analysis were studies that used ultrasound, performed by both vascular technicians and radiologists, to investigate occult vascular injuries. The composite data showed a positive ultrasound for injury to have a positive likelihood ratio (LR) of 35.4 (95% confidence interval (CI), 8.3–151) and a negative ultrasound to have a LR of 0.24 (95% CI, 0.08–0.72). Collectively, this data suggests that ultrasound is an appropriate first modality with which to investigate occult arterial injuries.

**CPC-EM Capsule**

What do we already know about this clinical entity?

*Acute vascular injury is a cause of disability. A detailed exam is key to diagnosis, however diagnosis remains difficult when soft signs of vascular injury are present*.

What makes this presentation of disease reportable?

*A physical exam demonstrating a strong radial pulse was misleading. Point-of-care ultrasound (POCUS) identified retrograde flow, which drew concern for arterial injury*.

What is the major learning point?

*When history and the physical exam conflict consider assessing the direction of flow with POCUS*.

How might this improve emergency medicine practice?

*It demonstrates that POCUS can assist and expedite the diagnosis of acute arterial injury and improve patient care*.

## CONCLUSION

Taken together, the current literature suggests that ultrasound is specific for vascular injuries, but may not be sensitive enough to rule out a vascular injury. If there is suspicion for vascular injury, despite a negative ultrasound, a CTA may be warranted. Careful ultrasonography, however, either by an emergency physician or a vascular technician, could potentially avoid unnecessary CTA in patients for whom the ultrasound shows strong evidence of vascular injury. Our patient had strong collateral flow from his ulnar artery, which masked the injury on physical examination alone. However, we were able to use the direction of flow on ultrasound to determine that this was in fact retrograde flow, indicating an injury to the proximal radial artery. Ultimately, determining the directionality of flow may improve the sensitivity of ultrasound, and, as in our clinical case, immediately inform the physicians that there is strong evidence of a vascular injury. This could allow emergency physicians and vascular surgeons to bypass CTA when appropriate to expedite clinical care through cost-effective use of diagnostic technologies.

## Supplementary Information

Video. The video clip illustrates color Doppler of the radial artery using point-of-care ultrasound in the transverse view. The scale in the lower left corner indicates that blood flowing toward the probe is represented with red, while blood flow away from the probe is represented with blue. The white arrow indicates the radial artery. The probe was angled in a proximal orientation towards the patient’s head. The blue color, within the radial artery, indicates blood flowing away from the transducer in a retrograde direction.
